# Embracing Large Language Models for Medical Applications, Part II: Building a Framework for Clinical Stewardship

**DOI:** 10.7759/cureus.115367

**Published:** 2026-08-28

**Authors:** Shiv Patil, Mert Karabacak, Matthew Southerby, Konstantinos Margetis

**Affiliations:** 1 Sidney Kimmel Medical College, Thomas Jefferson University, Philadelphia, USA; 2 Neurosurgery, Loma Linda University, Loma Linda, USA; 3 Radiotherapy Physics, University College London Hospitals NHS Foundation Trust, London, GBR; 4 Neurological Surgery, Mount Sinai Hospital, New York, USA

**Keywords:** artificial intelligence governance, clinical artificial intelligence, clinical validation, : large language models, medical informatics, patient safety

## Abstract

Large language models (LLMs) have moved rapidly from experimental demonstrations to early clinical, educational, and administrative use. The first phase of medical LLM discourse emphasized opportunities in transfer learning, domain adaptation, clinical decision support, education, privacy, fairness, and regulation. The next phase requires a more specific framework for clinical stewardship. Since 2023, medical LLMs have demonstrated substantial progress in medical question answering, diagnostic reasoning in interactive clinical dialogue, patient communication, documentation support, and retrieval-augmented knowledge synthesis. At the same time, prospective and randomized evaluations show that model performance alone does not guarantee improved physician reasoning, safer decisions, or better patient outcomes. The central challenge is therefore implementation: defining appropriate use cases, validating models in context, training clinicians, protecting patients, and monitoring deployed systems across their lifecycle. This Viewpoint proposes a practical roadmap for medical LLM adoption. We distinguish lower-risk administrative and communication tasks from higher-risk diagnostic, triage, and treatment tasks. We argue for tiered evidence standards, transparent reporting, local validation, human oversight, equity auditing, patient-centered consent, and continuous post-deployment surveillance. The regulatory environment has also matured, with risk-based frameworks and lifecycle-oriented oversight increasingly shaping how these systems are evaluated and maintained. LLMs should be treated as workflow-embedded sociotechnical systems, whose performance depends on the interaction among models, clinicians, patients, interfaces, local data, and governance. Responsible adoption will require collaboration among clinicians, clinical informaticians, health systems, patients, regulators, ethicists, and developers. Realizing the promise of medical LLMs depends on a shift from fascination with fluent outputs toward disciplined stewardship of clinical performance, accountability, equity, and trust.

## Editorial

In 2023, the case for large language models (LLMs) in medicine was framed around potential: models could summarize medical literature, support decision-making, improve patient education, and help clinicians navigate increasingly complex information environments. The article that initiated this discussion emphasized transfer learning, domain-specific fine-tuning, domain adaptation, reinforcement learning with expert input, dynamic training, interdisciplinary collaboration, education, validation, privacy, fairness, and regulatory frameworks [1]. While that framework remains relevant, the field has rapidly evolved: LLMs are no longer only a theoretical opportunity for medicine. They are now being evaluated in randomized trials, incorporated into ambient documentation platforms, explored for patient messaging, and optimized for medical question answering, diagnostic reasoning in interactive clinical dialogue, and multimodal reasoning [2-4].

The present question for medical leaders is therefore different. While the first phase asked how LLMs might be made medically useful, the next phase asks how medicine should govern, validate, teach, monitor, and limit these systems once they enter real clinical workflows. This shift matters because the most important risks are no longer confined to benchmark inaccuracy and can lead to actual clinical consequences. Such risks include automation bias, workflow distortion, privacy leakage, inequitable performance, unclear accountability, brittle retrieval, hallucinated confidence, insufficient consent, and institutional over-adoption before evidence is mature [5-7]. A clinically useful LLM must therefore be embedded in a responsible system. This concern is consistent with a recent SWOT analysis of artificial intelligence in clinical medicine, which highlighted that despite the reported benefits of AI, challenges related to data privacy, algorithmic bias, and the ongoing need for human oversight remain important considerations for ethical, transparent, and appropriate integration into clinical practice [8]. These risks are compounded by a reality that institutional governance does not fully reach: much of the real-world use of medical LLMs now occurs when patients consult general-purpose consumer tools directly, outside any clinical system.

From language models to clinical systems

The early public understanding of LLMs focused on text generation. In medicine, however, the more consequential development has been the transition from chatbot to clinical infrastructure. Contemporary systems can combine natural language, images, audio, structured records, laboratory results, waveforms, guidelines, and retrieved literature. Multimodal models have begun to integrate dermatologic images, electrocardiograms, clinical documents, and patient dialogue in simulated consultations [9,10]. Other models have been evaluated as agents that use tools, retrieve information, decompose questions, or support triage and referral workflows [11,12].

A primary example of large-scale deployment is the integration of generative AI tools within Epic, the market-leading electronic health record (EHR) platform. Epic has collaborated with ambient AI companies such as Abridge to integrate LLM-driven documentation solutions directly into the clinical workflow. In practice, these systems are capable of securely listening to the patient-clinician encounter, extracting relevant clinical details, and probabilistically synthesizing the unstructured dialogue into structured, specialty-specific draft notes. Epic has also integrated Augmented Response Technology (Art) for clinicians to automate note writing, patient message drafting, and hospitalization planning. The tool can also glean insights from chart data and prior results to identify imaging follow-ups, collect cancer staging details, and extract medication information. Microsoft’s Dragon Copilot is a similar tool that is being adopted to facilitate note documentation and reduce workflow burdens [13].

This expansion changes the clinical problem. A text-only model that answers a question incorrectly can mislead an individual user. In contrast, a multimodal or agentic system connected to clinical infrastructure can influence documentation, diagnostic possibilities, referrals, orders, patient messages, and institutional throughput. The unit of evaluation must therefore expand from answer accuracy to task safety, workflow reliability, clinician behavior, patient understanding, equity, and postdeployment performance. This expansion implies two distinct levels of evaluation that should not be conflated. Standalone, model-level evaluation, including benchmark accuracy, factuality, and reference-based scoring performed offline against a fixed test set, establishes whether a model's outputs are plausible, but it cannot establish whether the model helps or harms care once clinicians and patients act on those outputs. Embedded, workflow-level evaluation is therefore a separate and necessary layer: it requires prospective or randomized comparison of the clinician-model system against usual care, measured through downstream endpoints such as diagnostic and management decisions, time to escalation, documentation quality, patient comprehension, equity of performance across subgroups, and adverse events, together with postdeployment surveillance to detect drift. A model that performs well on the first axis should not be assumed to perform well on the second, and institutional or regulatory approval should require evidence from both levels before a system's autonomy is expanded. Medical LLMs should be evaluated as sociotechnical systems, meaning workflow-embedded tools whose safety depends on the model, interface, user, patient, institution, data environment, and governance process. Autonomy is a distinct dimension of this expansion. The risk a system poses depends not only on the stakes of the task but on how much the system is permitted to do without human action, ranging from drafting text a clinician must approve to placing orders or sending patient messages directly. Governing autonomy therefore requires explicit action permissions, human checkpoints at defined steps, reversibility of consequential actions, and constraints on the tools an agent may invoke.

Another emerging role for LLMs is the synthesis of patient-specific information into clinically useful summaries and suggestions. Prior work in neurosurgery has shown that structured ML models can support individualized risk estimation, prognostication, survival prediction, and clinically applicable phenotyping across conditions such as anterior lumbar interbody fusion, traumatic subdural hematoma, spinal cord glioma, and data-driven patient phenotypes [14-17]. LLMs could extend this paradigm by functioning as an interface layer that integrates structured model outputs with unstructured clinical notes, imaging reports, laboratory trends, guidelines, and patient preferences. Such systems should be evaluated as decision-support tools that generate patient-level syntheses for clinician review, with explicit safeguards against hallucination, automation bias, and unsupported recommendations.

A precise framing is preferable to a simple deterministic versus nondeterministic contrast. Structured predictive models usually depend on predefined features, labels, and outputs, whereas LLM-based systems probabilistically synthesize heterogeneous structured and unstructured information. In practice, LLMs may support clinician-facing orchestration around validated ML models, evidence retrieval, EHR data, and patient preferences. This role requires rigorous validation of both the underlying predictive models and the language-model interface that explains or contextualizes them.

Evidence has advanced yet remains uneven

Medical benchmarks were essential in the first phase of clinical LLM development. ChatGPT’s performance on licensing examinations drew attention to the possibility that general LLMs could reproduce medical reasoning patterns [18]. Med-PaLM and subsequent models such as Med-PaLM 2 showed that domain adaptation, instruction prompt tuning, and structured medical evaluation frameworks could substantially improve performance on medical question answering and long-form clinical responses, with physician-graded assessments approaching or matching physician-generated answers on some evaluation dimensions [2,3]. These studies were pivotal because they demonstrated that LLMs could encode clinically relevant knowledge and generate answers that physicians could interpret and evaluate along dimensions such as factuality, completeness, harm, and bias. These benchmarks have since become a weaker signal of clinical readiness: leading models now approach ceiling performance on licensing-style question sets, and concerns about test-set contamination make high scores difficult to interpret. Benchmark performance alone is no longer a sufficient metric for clinical value. Static question sets cannot capture several limitations that determine real-world usefulness: possible test-set contamination from public licensing materials appearing in training corpora, the absence of external, multisite validation for most published benchmarks, and a limited and inconsistent correlation between benchmark accuracy and real-world clinical outcomes such as diagnostic yield, time to correct management, or harm avoided. Because of these limitations, the field should treat benchmark scores as a coarse screening step at most and should weight prospective, workflow-based, and patient-centered evaluation, the kind described throughout this Viewpoint, far more heavily than leaderboard performance when judging clinical value.

While test-based medical question answering is static, real-world clinical reasoning unfolds over time. Physicians generate and revise differential diagnoses, test assumptions, gather missing information, communicate uncertainty, consider patient preferences, and decide when inaction is safer than intervention. Recent evaluations show that LLM performance can vary across these stages. A 2026 cross-sectional study of frontier models found stronger performance in final diagnosis and management than in differential diagnosis and uncertainty-rich early reasoning [19]. Randomized studies also show that providing physicians with GPT-4 access does not automatically improve diagnostic reasoning compared with conventional resources [5]. A related randomized trial found improved performance on management reasoning tasks with GPT-4 assistance, highlighting that effects may depend heavily on the task, user, interface, and evaluation design [20]. Taken together, these analyses reveal that the combination of a clinician and a model can perform worse than the model alone, a pattern often described as the problem of human-AI complementarity. The objective of deployment is therefore not simply to field a capable model but to engineer a clinician-model system that outperforms either party working alone, which depends as much on interface, workflow, and training as on the model itself.

The practical implication is that model capability and clinical benefit must be separated. A model can perform well in isolation while failing to improve care when used by clinicians under time pressure. Conversely, a modest model may be useful when placed in a carefully bounded workflow, such as summarizing a visit or drafting an inbox response that a clinician edits. Medical LLM adoption should therefore prioritize prospective evaluation, workflow-specific outcomes, human factors, and postdeployment surveillance over leaderboards. Prospective evidence must also be read critically. Much of the published literature on clinical LLMs comes from single sites, relies on surrogate endpoints such as user satisfaction, or is produced or funded by the vendors whose products are being evaluated, while independent, adequately powered, prospective studies remain comparatively scarce.

LLMs as workforce and knowledge infrastructure

Clinical LLMs should also be considered in the context of growing workforce pressure and expanding biomedical information. For example, workforce modeling in neurosurgery projects that demand will substantially outpace supply by 2037, with national adequacy declining under both status quo and improved-access scenarios [21]. At the same time, specialty literature is expanding at a scale that challenges traditional manual synthesis. These pressures suggest that one important role for LLMs may be to extend clinicians' cognitive capacity by summarizing records, retrieving current evidence, preparing focused literature syntheses, and generating patient-specific summaries for clinician review. Such systems should be developed as assistive infrastructure, with source attribution, uncertainty communication, workflow-specific validation, and monitoring for hallucination, bias, and over-reliance.

Prioritizing use cases by clinical risk

The next stage of LLM adoption should distinguish tasks by risk, evidence requirements, and expected benefit. Lower-risk, high-value uses include draft documentation, visit summarization, chart abstraction, patient education at appropriate reading levels, translation support with review, administrative letters, coding assistance, literature summarization, and inbox response drafting. These uses can reduce burden while preserving clinician review before information reaches the chart or patient. Patient communication studies suggest that LLM-generated responses can be rated highly for quality and empathy, although length, readability, context, and clinical correctness remain important concerns [22,23]. Patient education is another high-yield, lower-risk use case when clinician review and quality controls are preserved. A scoping review of 201 studies identified major LLM applications in generating patient education materials, interpreting medical information, providing lifestyle recommendations, supporting customized medication use, offering perioperative instructions, and optimizing doctor-patient interaction [24].

Intermediate-risk uses include previsit chart synthesis, guideline retrieval, clinical trial matching, referral prioritization, tumor board preparation, structured radiology or pathology reporting, and medication reconciliation support. These applications affect clinical decisions indirectly, so they require local validation, clear source attribution, and escalation rules when outputs are uncertain or incomplete. Retrieval-augmented generation may help ground responses in approved sources, but retrieval quality, source currency, and citation faithfulness must be validated rather than assumed [25,26].

Higher-risk uses include autonomous triage, diagnosis, treatment recommendations, medication dosing, discharge readiness, patient-facing symptom assessment, and unsupervised decision support. These tasks require stronger evidence, including silent, noninterventional testing in real workflows before outputs are shown to clinicians or patients, prospective studies, randomized trials where feasible, subgroup analyses, and monitoring after deployment. A tiered approach lets health systems gain benefit from safer applications while resisting premature deployment in areas where errors could directly harm patients. Medication guidance is a higher-stakes patient-facing use case. LLMs may help explain drug interactions, side effects, and emergency protocols, yet the risk of misinformation and lack of patient-specific context require clinician involvement, regulatory oversight, and clear escalation pathways [24].

Ambient documentation as the first large-scale test

Ambient documentation is one of the most visible early clinical applications of generative AI. These systems listen to clinical encounters, create draft notes, and allow clinicians to edit documentation before signing. Early studies report potential improvements in documentation burden, clinician experience, and perceived usability [27,28]. This use case is attractive because it addresses a widely recognized contributor to burnout and may allow clinicians to maintain more eye contact and attention during visits.

Ambient documentation also illustrates why lower-risk applications still require governance. AI-generated notes may omit relevant negatives, over-normalize patient language, insert unsupported details, distort temporality, or create medicolegal ambiguity if clinicians review them too quickly. They may also generate verbose or formulaic notes that fail to reflect the clinician's usual documentation style, specialty-specific reasoning, or preferred level of detail. Consent practices are variable, and patients may misunderstand whether the tool records, stores, analyzes, or reuses their conversation [29]. Documentation tools should therefore include explicit patient-facing explanations, easy opt-out procedures, clinician editing requirements, audit logs, quality assurance sampling, specialty-specific templates, clinician-level customization, and monitoring for note bloat as well as factual errors. They should also be evaluated for note accuracy, concision, clinician time, patient trust, billing effects, and differential performance across specialties, languages, accents, and visit types.

Ambient documentation should be viewed as a valuable but partial pilot domain for broader clinical LLM governance. It tests the same issues that will appear in higher-risk tools: disclosure, privacy, usability, review burden, accuracy, drift, equity, liability, and institutional accountability. This analogy, however, has a real limit: ambient scribes primarily summarize and extract from a bounded conversation, a task that does not exercise the failure modes distinctive to autonomous reasoning or agentic action, such as multistep hallucinated reasoning chains, tool-use errors, or automation bias embedded in a high-stakes recommendation. Governance experience from documentation tools should therefore inform but cannot substitute for dedicated evaluation frameworks for diagnostic, triage, and treatment-recommendation systems, which must be tested under uncertainty, adversarial inputs, and multistep decision chains that ambient scribing does not exercise.

Reporting, validation, and lifecycle monitoring

Version 2 of medical LLM adoption should be anchored in transparent reporting and lifecycle evaluation. Transparent Reporting of a Multivariable Prediction Model for Individual Prognosis or Diagnosis-Large Language Models (TRIPOD-LLM) provides a reporting framework for studies that develop, tune, prompt engineer, or evaluate LLMs in biomedical contexts [30]. TRIPOD+AI, Consolidated Standards of Reporting Trials-Artificial Intelligence (CONSORT-AI), Standard Protocol Items: Recommendations for Interventional Trials-Artificial Intelligence (SPIRIT-AI), Developmental and Exploratory Clinical Investigations of Decision Support Systems Driven by Artificial Intelligence (DECIDE-AI), and, for imaging-focused or multimodal imaging studies, Checklist for Artificial Intelligence in Medical Imaging (CLAIM) provide complementary reporting expectations for prediction models, clinical trials, protocols, early-stage clinical evaluation of AI decision-support systems, and medical imaging AI studies [31-35]. These frameworks should be adapted pragmatically to LLM studies. Multimodal conversational systems that combine imaging, dialogue, and structured data pose a particular reporting gap: CLAIM was developed for imaging-focused prediction models and does not fully capture elements specific to multimodal LLMs, such as cross-modal consistency, modality-specific error and hallucination rates, or how conflicting signals across modalities are resolved. Until reporting guidelines catch up with this use case, studies of multimodal clinical LLMs should report performance and failure modes separately by modality and by modality combination, in addition to aggregate results.

At minimum, clinical LLM reports should specify the model name and version, date of access, input data, prompt strategy, retrieval sources, output format, temperature or generation settings when available, evaluator qualifications, patient population, task definition, comparator, failure modes, subgroup results, human oversight, and intended use. These reporting expectations are straightforward for open-weight or static models but harder to operationalize for proprietary, continuously updated commercial systems, where the exact model weights, training data composition, and sometimes even a granular version identifier are not disclosed by the vendor. In these cases, reports should specify the model name and vendor-designated version string, the exact date range of API or interface use, any known deployment or system-prompt changes communicated by the vendor during the study period, and, where possible, retained input-output transcripts or a frozen snapshot of representative outputs so that findings remain interpretable even after the underlying model can no longer be queried in its original state. Authors should also explicitly disclose when a vendor does not guarantee version stability during a study period, since this materially affects reproducibility and should be reported as a limitation rather than omitted. Studies should report more than accuracy: relevant outcomes include calibration, uncertainty handling, harmful omissions, hallucinated facts, inappropriate reassurance, escalation performance, equity, time burden, clinician acceptance, patient comprehension, and downstream utilization.

Lifecycle monitoring is equally important. LLMs change through vendor updates, prompt modifications, retrieval database updates, EHR integration changes, and shifts in clinical practice. A model validated in January may behave differently later in the year. Health systems should maintain version control, audit trails, incident reporting pathways, drift surveillance, revalidation triggers, and decommissioning criteria. Evaluation is therefore a continuing clinical safety process rather than a one-time procurement step. Versioning also bears on the scientific record. Because vendors retire and replace models, a study evaluating a specific version may become impossible to reproduce once that version is withdrawn, which argues for precise version reporting and, where feasible, retention of the evaluated model or its outputs.

Regulatory frameworks have begun to catch up with these expectations. In the United States, the Food and Drug Administration finalized guidance in 2025 on predetermined change-control plans, which let manufacturers specify in advance the modifications an AI-enabled device may undergo and the methods for validating them, an approach that aligns closely with the lifecycle monitoring described here; current change-control guidance, however, was developed primarily for conventional machine-learning models and does not yet fully address generative systems. This lifecycle guidance also does not resolve a more fundamental regulatory question that is especially salient for LLMs: whether a given application qualifies as Software as a Medical Device (SaMD) at all. Under the 21st Century Cures Act, clinical decision-support software is exempted from FDA device regulation when it is not intended to analyze medical images, signals, or patterns and when it is intended to allow a clinician to independently review the basis for its recommendations rather than rely on the software's output as the primary basis for a clinical decision. Many LLM applications are designed explicitly to satisfy this exemption by presenting information for clinician review rather than issuing directive outputs, yet the requirement that a clinician be able to independently review the basis for a recommendation is difficult to apply to a probabilistic, nondeterministic system whose internal reasoning cannot be fully inspected. This ambiguity means that a substantial share of deployed LLM functionality may fall into a regulatory gray zone between SaMD and exempt clinical decision support, leaving oversight to depend heavily on local governance rather than federal device regulation. In the European Union, the AI Act classifies AI used for diagnosis, clinical decision support, treatment recommendation, triage, and patient monitoring as high risk, with obligations for high-risk systems phasing in from 2026 and an extended transition for software already regulated as a medical device. The World Health Organization has issued dedicated ethics and governance guidance for large multimodal models in health. Health systems should treat these frameworks as a floor rather than a ceiling, recognizing that much current LLM functionality is deployed as clinical decision support that may fall outside device regulation and therefore depends on local governance for safe use.

Safety, trust, and knowledge integrity

LLMs generate fluent text, which can create an illusion of certainty. In medicine, this fluency is both useful and dangerous. Hallucinated citations, fabricated mechanisms, overconfident diagnoses, and ungrounded treatment suggestions can be difficult for patients and busy clinicians to detect. Clinician-facing systems should therefore communicate uncertainty, cite verifiable sources when evidence is retrieved, and facilitate comparisons of outputs with institutional guidelines. Patient-facing systems should use conservative safety thresholds, provide clear escalation instructions, and reinforce timely clinical assessment when symptoms, risk factors, or uncertainty warrant human care.

Knowledge integrity requires more than retrieval. Medical LLMs may be exposed to large, heterogeneous, and inconsistently vetted training corpora, contaminated evaluation sets, or deliberately poisoned data. A study of data poisoning found that small amounts of medical misinformation in training data could increase harmful outputs while corrupted models could still perform well on common benchmarks [6]. This finding further demonstrates that benchmark performance alone cannot serve as a primary safety signal. Developers and health systems should test for adversarial misinformation, evaluate outputs against biomedical knowledge bases, monitor hallucination rates, and build layered safeguards around high-risk content. The threat model extends beyond training-time corruption to the point of use. Systems that retrieve documents or act as agents are vulnerable to indirect prompt injection, in which adversarial instructions embedded in a retrieved web page, a clinical note, or other ingested content alter the model’s behavior; for deployed retrieval and agentic tools, this inference-time risk is often more pressing than data poisoning. A related operational hazard is unsanctioned use, in which clinicians paste protected health information into consumer tools outside institutional control. Mitigations include sandboxing and provenance checks on retrieved content, sanctioned and access-controlled tooling, and contractual data-handling protections such as business associate agreements.

Retrieval-augmented generation is one strategy for grounding outputs in curated sources, especially when source currency, institutional guidance, or citation traceability matter. Longer-context models may reduce the need for retrieval in some bounded tasks by allowing more source material to be included directly in the prompt. However, both approaches have failure modes. Retrieval can miss relevant documents, overlook information distributed across sources, or cite material that only weakly supports the generated claim. Long-context approaches can still struggle when important information is sparse, contradictory, or difficult to locate within a large input. Clinical systems should therefore evaluate retrieval-based, long-context, and hybrid approaches end to end, including source selection, source interpretation, citation accuracy, and clinician usability [25,26].

Evidence synthesis illustrates both the promise and the limits of medical LLMs. The biomedical literature has expanded beyond what individual clinicians can reliably follow: in neuro-oncology, neuro-linguistic programming (NLP)-based topic modeling of 43,329 glioblastoma publications has shown that computational methods can map large research domains, identify emerging trends, and guide future inquiry [36]. However, systematic reviews require methodological judgment that extends beyond retrieval and summarization, including formulation of clinically meaningful questions, assessment of study relevance and bias, interpretation of heterogeneous findings, and responsibility for conclusions. Sarkar, writing from the perspective of Cochrane, argues that current AI tools remain unsuitable for autonomous production of high-quality scientific reviews, although they may help structure data, manage large evidence volumes, detect retractions or problematic studies, and support human reviewers [37]. Literature synthesis should therefore require study-level traceability, source verification, citation fidelity checks, transparent reporting of AI use, and expert human oversight. AI-assisted evidence synthesis should preserve human methodological judgment because systematic reviews inform clinical practice, public health guidance, and policy decisions where errors can mislead care or waste resources.

Mechanistic interpretability to enhance LLM legitimacy

A further safeguard, distinct from the retrieval and provenance checks discussed above, is mechanistic interpretability: the effort to uncover the internal computations of LLM inference as human-interpretable circuits so that, as model capabilities advance, their safety, alignment, and behavior can still be assessed and controlled [38]. This matters for clinical deployment because several of the failure modes raised throughout this Viewpoint, including hallucinated confidence, sociodemographic bias, automation bias, and opaque agentic behavior, are currently detected only at the level of outputs. Interpretability offers a route to understanding why a model produces a given output, rather than only auditing whether that output was correct.

The subfield has begun to show that this is tractable. Sparse autoencoders have been used to recover human-interpretable features from neuron activations [39], and activation engineering has been shown to be capable of steering models' behavior, including making a model act as though it believes itself to be deployed versus evaluated [40]. In clinical contexts specifically, sparse autoencoder-based approaches have been proposed to reveal internal model features and identify failure modes relevant to deploying LLMs as cognitive aids [41], and at least one study has directly applied mechanistic interpretability methods to characterize variability in clinical reasoning across medical LLMs [42]. These findings are encouraging, but they should be read against an important limitation: most mechanistic interpretability techniques, including sparse autoencoders, have so far been validated primarily on smaller or research-scale models and on relatively constrained tasks, and scaling them to the massive, frequently updated, multibillion-parameter frontier models now being deployed clinically remains an unsolved engineering challenge. Feature dictionaries and circuits identified in one model or model checkpoint may not transfer to another, and the computational cost of applying these methods grows substantially with model size. Interpretability should therefore be understood as a promising but currently limited-scale safeguard, not yet a scalable substitute for the prospective and equity evaluation described elsewhere in this Viewpoint.

These early results suggest plausible clinical applications beyond diagnostic reasoning, including the identification of circuits associated with sociodemographic bias, hallucination, and data security vulnerabilities: failure modes this Viewpoint has identified as central to safe deployment. However, the field has not yet demonstrated reliable extraction of causal circuits underlying complex outputs such as a specific diagnosis or treatment recommendation, and claims to that effect should be treated as a goal for the field rather than a current capability. As with benchmark performance, interpretability findings should not be mistaken for clinical validation.

Given how early this work remains, the priority is to apply existing mechanistic interpretability techniques to clinical risk models now rather than waiting for the methods to mature in isolation from clinical use. Doing so would both ground the interpretability field in clinically meaningful problems and give health systems an additional, mechanism-level form of evidence, alongside the prospective and equity evaluations described above, for deciding when a model's outputs can be trusted in each workflow.

Equity as a core safety requirement

Equity is a core safety requirement and a primary measure of clinical AI quality. Prior healthcare algorithms have shown how biased labels, unequal access, and proxy outcomes can reproduce inequity at scale [43]. LLMs add additional risks because they are trained on large, heterogeneous datasets that may encode stereotypes, underrepresent certain populations, and perform differently across languages, dialects, literacy levels, disability groups, and cultural contexts. Bias in an LLM may affect diagnostic framing, pain interpretation, triage advice, empathy, referral suggestions, or the reading level of patient education materials.

Health systems should require subgroup evaluation before deployment and ongoing equity monitoring afterward. Relevant dimensions include race and ethnicity, sex and gender, age, disability, pregnancy status, socioeconomic context, insurance status, rurality, language, literacy, and local disease prevalence. Evaluation should include patients and community representatives because model outputs can be technically accurate while still being culturally unsafe, confusing, stigmatizing, or impractical. Recent calls to address AI algorithmic bias emphasize shared responsibilities among healthcare organizations, developers, and regulators [44]. For medical LLMs, shared responsibility should mean that equity data, failure modes, and mitigation plans are visible to the institutions and patients affected by deployment. However, a tension runs through these requirements. The settings that might benefit most from language models (i.e., those facing the most severe clinician shortages and the greatest barriers to specialist knowledge) are frequently the least able to fund the local validation, subgroup analysis, and continuous monitoring that responsible deployment demands. Acknowledging this asymmetry is necessary so that stewardship does not become a mechanism that concentrates the benefits of these tools in well-resourced systems.

Operationalizing subgroup evaluation requires more specific criteria than a general commitment to fairness. At minimum, predeployment testing should stratify performance by the dimensions listed above using sample sizes adequate to detect clinically meaningful differences and should report both aggregate and subgroup-specific metrics for accuracy, omission of relevant findings, hallucination rate, and, for patient-facing tools, comprehension and trust. Health systems should predefine an acceptable performance gap before testing begins, for example, a maximum absolute or relative difference in error or harmful-omission rates between the best- and worst-performing subgroups, rather than judging disparities after the fact. Any subgroup falling outside that threshold should trigger mitigation or a delay in deployment rather than a footnote in a validation report. Where subgroup sample sizes are too small at a single institution to support this kind of analysis, shared validation across institutions or registries (discussed further below) becomes necessary rather than optional. These thresholds will not be identical across use cases: a modest disparity in a low-risk administrative tool carries different implications than the same disparity in a triage or treatment-recommendation system, and the tiered risk framework proposed earlier in this Viewpoint should set the bar accordingly.

Disease-specific disparities in health literacy and access provide a concrete rationale for evaluating LLMs across literacy and socioeconomic strata. In a national intracranial tumor cohort, Hispanic/Latino and non-Hispanic Black patients reported greater difficulty understanding medical information and completing health-related forms, as well as greater financial and transportation barriers [45]. LLMs may help translate complex information into accessible language, yet their outputs must be validated across languages, literacy levels, cultures, and care-access contexts.

Clinician education and professional identity

Medical education must evolve with clinical AI. Early literature on LLMs in medical education described opportunities for curriculum development, tutoring, question generation, exam preparation, and personalized learning [46,47]. Medical education literature also supports using generative language models for realistic simulations, digital patients, personalized feedback, evaluation methods, and reducing language barriers [48]. Version 2 of LLM adoption should define AI literacy as a core clinical competency. Clinicians need to understand how LLMs generate outputs, why they hallucinate, how prompting affects performance, when retrieval helps, how automation bias appears, and how to document AI-assisted work.

This training should be practical. Clinicians should learn to ask LLMs for uncertainty, alternatives, citations, contraindications, and missing information. They should learn to recognize plausible but unsupported explanations. Training should specify situations in which LLM use is inappropriate, including emergencies, legally sensitive communications, and encounters requiring nuanced physical examination. Medical trainees need special guidance because overuse may weaken independent reasoning. LLMs can be powerful tutors when they ask learners to justify decisions, critique differentials, and compare evidence. They can also become cognitive shortcuts if used to bypass the difficult work of clinical synthesis. Concern about deskilling is not hypothetical. In a multicenter study of experienced endoscopists, routine use of an AI detection aid was followed by a decline in adenoma detection during unassisted colonoscopy, from 28.4% to 22.4%, an absolute reduction of roughly six percentage points [49]. The finding suggests that sustained reliance on assistive AI can erode unaided performance, with direct implications for how trainees and practicing clinicians should use these systems.

Professional identity will change. Clinicians will increasingly supervise machine-generated drafts, decide whether an output is safe, and explain AI-assisted processes to patients. This supervisory role requires time, accountability, and institutional support. Oversight should be substantive, documented, and feasible within the clinical workflow.

Accountability, consent, and patient trust

Patients should be able to understand when AI is involved in their care and what role it plays. The level of disclosure may vary by use case. A clinician using an LLM privately to simplify a patient education handout differs from an ambient scribe recording an encounter or a triage tool advising a patient where to seek care. More direct patient interaction, greater data capture, and higher clinical stakes require clearer consent and disclosure.

Accountability must also be explicit. For clinical documentation, the signing clinician remains responsible for the note. For decision support, the institution must define who reviews the output, how disagreements are handled, and how errors are reported. For patient-facing tools, escalation pathways and emergency instructions must be built in. For tools connected to the EHR, audit logs should record inputs, outputs, model version, and final human action when appropriate. These records should support quality improvement, medicolegal review, and detection of systematic failures. Accountability also intersects with evolving legal standards. As AI assistance becomes common, questions arise about whether failing to use an available tool or relying on one uncritically could bear on the standard of care and about how responsibility is apportioned among the clinician, institution, and developer under professional-liability and product-liability principles. These questions remain unsettled, but health systems should anticipate them when defining oversight, documentation, and disclosure practices.

Trust will depend on visible safeguards. Patients and clinicians need assurance that tools are locally validated, monitored, privacy-preserving, equitable, and limited to appropriate roles. Health systems should resist the temptation to present LLMs as magical general intelligence. They should describe them as assistive technologies with defined uses, known limitations, and accountable human oversight.

Direct-to-consumer use and the limits of institutional stewardship

Much of the framework above presumes that an institution selects, validates, and monitors a tool. Yet the largest share of real-world medical LLM use occurs outside this model, when patients consult general-purpose consumer assistants directly, with no clinical validation, disclosure, or oversight. Institutional stewardship cannot govern this use, but it cannot ignore it either. Clinicians increasingly encounter patients who arrive having already obtained an interpretation, a differential, or a treatment suggestion from a consumer tool, and the clinical task shifts toward eliciting what the patient was told, correcting misunderstandings, and contextualizing generic output for the individual. Health systems can respond by preparing clinicians to engage with this material constructively rather than dismissively, by offering vetted patient-facing resources and, where appropriate, sanctioned tools that meet basic quality and privacy standards, and by communicating plainly about what consumer systems can and cannot safely do. A stewardship agenda that addresses only institution-deployed systems will leave the most common form of patient exposure unmanaged.

Institutional implementation

A health system preparing to deploy an LLM should create an institutional pathway that resembles medication formulary review or device governance. The first step is an intended-use statement that names the clinical task, user, data inputs, output, risk level, patient population, and prohibited uses. The second step is predeployment testing with local cases, including edge cases, underrepresented groups, and realistic workflow constraints. The third step is controlled release with training, monitoring, feedback capture, and predefined stop criteria. The fourth step is periodic revalidation after model updates, prompt changes, retrieval database changes, or changes in evidence.

This process should include clinicians from affected specialties, clinical informaticians, privacy officers, patient representatives, quality leaders, and risk management. Procurement should require model documentation, data-handling terms, update notifications, performance evidence, and mechanisms for incident investigation. Implementation science is essential because poor interface design can convert a technically capable model into an unsafe tool. Conversely, a well-bounded model with clear escalation rules may provide value even when its standalone benchmark performance is modest. The goal is to make LLM deployment observable, auditable, and capable of being paused, rolled back, or retired when safety signals emerge.

It is useful to frame this function as clinical stewardship in the established sense of the term. Just as antimicrobial stewardship pairs access to powerful agents with an accountable program governing indication, selection, duration, and monitoring, AI stewardship implies a standing multidisciplinary body, an institutional formulary of approved uses, explicit indications and prohibited uses, and mandatory surveillance. This framing also exposes a practical tension. The validation, monitoring, and revalidation expected for each tool is substantial, and vendors are introducing LLM features faster than most institutions can independently evaluate them, which risks either paralysis or perfunctory approval. Shared validation across institutions, registries of deployed tools and their performance, and governance calibrated to risk can ease this burden, and the appropriate level of oversight is proportionate rather than maximal, since excessive friction and delayed access to beneficial tools also carry costs for patients.

An updated 10-point agenda

The original 10 suggestions for LLMs in medicine remain a useful foundation [1]. Version 2 should recast them around clinical stewardship (Table 1). First, define the intended use, user, patient population, risk level, and degree of autonomy before deployment. Second, begin with lower-risk, high-burden workflows where clinician review is preserved and burden reduction is measured against clinical reasoning, equity, and patient safety. Third, require local validation using data, patients, languages, and workflows that match the deployment setting. Fourth, evaluate models across the clinical reasoning continuum, including information gathering, differential diagnosis, uncertainty handling, management planning, patient communication, and follow-up. Fifth, use reporting standards such as TRIPOD-LLM, CLAIM for imaging-focused or multimodal studies, and AI-specific trial guidelines, with traceability and citation verification for evidence-synthesis use cases, and align deployments with applicable regulatory frameworks such as the FDA predetermined change-control pathway, the EU AI Act, and WHO guidance. Sixth, monitor performance after deployment with drift detection, incident review, and revalidation after updates. Seventh, treat equity, accessibility, and language performance as safety metrics. Eighth, train clinicians and trainees in AI literacy, verification, documentation, and patient communication. Ninth, create patient-centered policies for disclosure, consent, privacy, and opt-out. Tenth, make accountability auditable by preserving human oversight, version control, and clear responsibility for final clinical action.

**Table 1:** Ten updated suggestions for implementing LLMs in medicine LLM: large language model.

No.	Suggestion
1	Define intended use, accountable user, patient population, clinical risk, and degree of autonomy before deployment.
2	Prioritize lower-risk, high-burden workflows with mandatory clinician review, and evaluate whether LLMs reduce cognitive, documentation, and information-management burden without degrading clinical reasoning, equity, or patient safety.
3	Perform local validation using representative patients, languages, specialties, and workflows, including validation of patient-level syntheses against source data, predictive-model outputs, and clinician judgment before clinical use.
4	Evaluate the full reasoning continuum, including information gathering, differential diagnosis, uncertainty handling, management planning, patient communication, and follow-up.
5	Report studies using transparent LLM and AI reporting standards, with study-level traceability, citation verification, disclosure of AI use, and human methodological oversight for LLM-assisted evidence synthesis, and align with applicable regulatory frameworks (FDA predetermined change control, EU AI Act, WHO guidance).
6	Monitor deployed systems for drift, errors, bias, workflow effects, version changes, interpretability, and failure modes.
7	Treat equity, accessibility, language performance, health literacy, socioeconomic context, and care-access barriers as safety metrics.
8	Train clinicians and trainees in AI literacy, verification, documentation, and patient communication.
9	Use patient-centered disclosure, consent, privacy, and opt-out policies.
10	Preserve auditable human oversight, version control, clear accountability for final clinical action, and the ability to pause, roll back, or retire unsafe deployments.

This agenda is intentionally practical. It does not require every LLM application to undergo the same evidentiary pathway. It requires evidence proportional to risk. Drafting an administrative letter, suggesting a differential diagnosis, and autonomously advising a patient with chest pain should never be governed as equivalent tasks. Medicine needs flexible governance that supports beneficial innovation while protecting patients from premature generalization.

Conclusion

The next version of medical LLM adoption should be defined by stewardship. LLMs have demonstrated impressive progress in medical knowledge, dialogue, documentation, and communication. They may reduce clerical burden, broaden access to understandable medical information, and support clinicians overwhelmed by data. Yet their risks are inseparable from their strengths: fluency can mask error, scale can amplify bias, integration can spread failure, and convenience can encourage over-reliance. The path forward is disciplined clinical integration. Health systems should choose use cases carefully, validate locally, monitor continuously, educate users, disclose appropriately, and keep patients at the center. Developers should design for uncertainty, verifiability, equity, and auditability. Clinicians should retain final judgment while learning to supervise AI outputs responsibly. Researchers should move beyond benchmark scores toward prospective, workflow-aware, patient-centered evidence. Above all, the goal is not a capable model but a capable system: clinician and machine together achieving what neither achieves alone.
